# Aortic plaque morphology and cholesterol crystal release assessed using non-obstructive general angioscopy

**DOI:** 10.1016/j.athplu.2026.100623

**Published:** 2026-11-07

**Authors:** Yudai Tanaka, Keisuke Kojima, Kohei Agune, Tokio Nishiwaki, Naoki Kurito, Satoaki Tomimatsu, Yuta Hotsubo, Tetsuro Nagao, Yuki Nakajima, Ran Sumida, Koichiro Hori, Saki Mizobuchi, Shohei Migita, Masatsugu Miyagawa, Yutaka Koyama, Katsunori Fukumoto, Yasunari Ebuchi, Riku Arai, Suguru Migita, Mitsumasa Sudo, Daisuke Kitano, Daisuke Fukamachi, Yasuo Okumura

**Affiliations:** Division of Cardiology, Department of Medicine, Nihon University School of Medicine, Tokyo, Japan

**Keywords:** NOGA, Cholesterol crystals, Aortic plaques, Puff chandelier rupture

## Abstract

**Background:**

Non-obstructive general angioscopy (NOGA) enables direct visualization and dynamic assessment of aortic plaque morphology and surface disruption, compared with conventional imaging modalities such as computed tomography. Aortic plaque disruption has been implicated in organ injury, potentially through the release of cholesterol crystals (CCs) into the bloodstream and subsequent inflammatory responses in peripheral organs. However, differences in CC detection according to aortic plaque morphologies have not been fully investigated.

**Methods:**

We prospectively enrolled 144 consecutive patients with coronary artery disease who underwent NOGA between September 2021 and December 2024. A total of 350 aortic plaque sites in the infrarenal abdominal aorta were classified into six morphological types and analyzed. The presence of CCs was assessed using a filter paper rinse method under polarized light microscopy, and detection rates were compared across plaque morphologies.

**Results:**

CCs were detected in 79 of 350 plaque-site samples (22.6%). CC detection differed significantly across plaque morphologies and was highest in puff chandelier ruptures (42.6%), followed by puff ruptures (16.3%), thrombi (2.9%), ulcers/fissures (2.2%), and yellow plaques (1.5%). After accounting for within-patient clustering using a mixed-effects logistic regression model with a patient-specific random intercept, plaque morphology was significantly associated with CC detection (global P < 0.0001). In patient-level multivariable analyses, the number of puff chandelier ruptures remained independently associated with CC detection after adjustment for relevant comorbidities and laboratory parameters.

**Conclusion:**

Puff chandelier rupture was associated with the highest rate of CC detection, suggesting that this morphology may represent a biologically active plaque phenotype with greater CC shedding potential. These findings link direct plaque morphology to a functional marker of plaque activity.

## Highlights

• Cholesterol crystal detection varies markedly according to aortic plaque morphology.
• Puff chandelier rupture shows the highest cholesterol crystal detection rate.
• Puff chandelier rupture may represent a biologically active plaque phenotype.

## Graphical abstract

**Figure:**
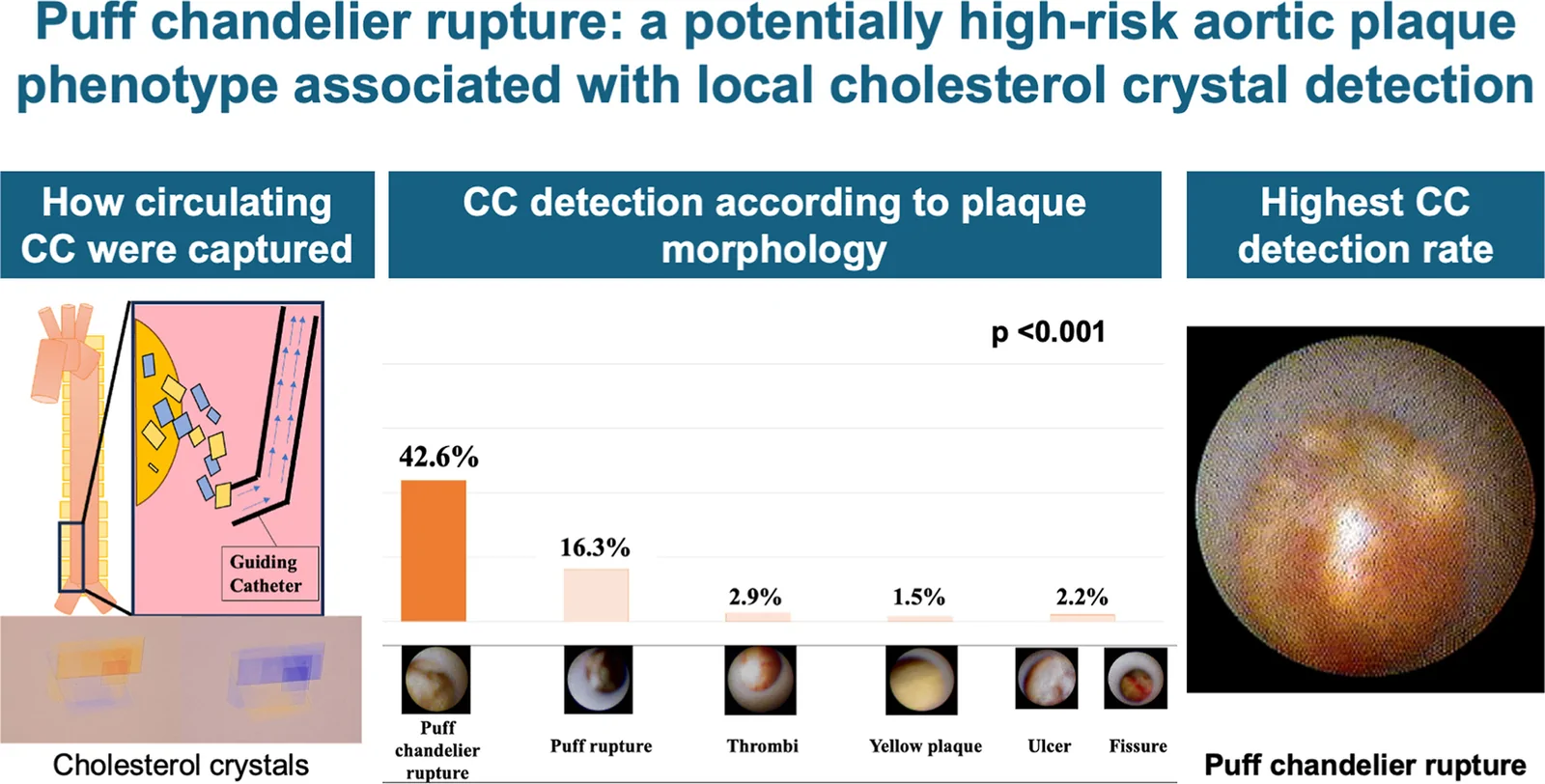

## 1 Introduction

Atherosclerosis is the underlying pathophysiology of life-threatening diseases such as coronary artery disease (CAD) and stroke, and it also affects the aorta, the largest artery in the body [1]. Advanced aortic atherosclerosis contributes not only to conditions like aortic dissection and aneurysm but also to systemic embolism and serves as a marker of systemic atherosclerotic burden [2]. Previous studies have shown associations between aortic plaque burden and risks of cerebral infarction and lower limb ischemia [3] [4].

Non-obstructive general angioscopy (NOGA) provides high-resolution, dynamic visualization of aortic plaques and has demonstrated that plaques may appear ruptured even in aortic segments deemed normal on CT [2] [5] [6] [7] [8]. Previous NOGA studies, including the EAST-NOGA study, have primarily characterized the burden and distribution of aortic plaques and demonstrated associations between overall plaque burden and subsequent cardiovascular events [9]. However, these previous studies did not systematically and quantitatively determine whether specific macroscopic plaque morphologies differ in their association with local CC detection.

In coronary arteries, thrombus-forming vulnerable plaques are typically defined by rupture or erosion, whereas the aorta's large diameter makes thrombotic occlusion less common [10] [11]. Instead, the biological activity of aortic plaques may be reflected by CC detection in the circulation or by their potential for CC shedding. Therefore, the present study aimed to systematically and quantitatively evaluate the association between predefined NOGA-derived aortic plaque morphologies and CC detection in locally sampled blood in a consecutive cohort. By directly linking macroscopic plaque morphology with an in vivo functional marker related to plaque activity and CC shedding potential, this study sought to establish a morphology–function relationship in aortic atherosclerosis.

## 2 Methods

The data, analytic methods, and study materials will be made available from the corresponding author on reasonable request for purposes of reproducing the results.

### 2.1 Study design and subjects

This was a single-center, prospective, observational study of 144 consecutive patients with CAD who underwent NOGA for the aorta at Nihon University Itabashi Hospital between September 2021 and December 2024. Patients were eligible for inclusion if they were aged over 20 years and underwent coronary angiography or percutaneous coronary intervention (PCI). Patients were enrolled at the discretion of the attending physicians. Exclusion criteria were identical to those of our previous study [9], including the use of mechanical circulatory support, hemodynamic instability (ie, Killip class >2 or cardiogenic shock), acute aortic syndrome, acute-phase cerebral infarction, allergy to contrast media, and pregnancy. The study received approval from the institutional review board at Nihon University Itabashi Hospital (RK-261013-14) and was registered in the University Hospital Medical Information Network Clinical Trials Registry of Japan (UMIN000051008). The requirement for individual informed consent was waived by the Institutional Review Board, and the study was conduced using an approved opt-out consent procedure. Information regarding the study was made publicly available, and patients were provided with an opportunity to decline participation.

### 2.2 Cardiac catheterization and angioscopic system

Following the same procedure as outlined in the previous study [9], cardiac catheterizations were carried out using a standard approach via either the radial or femoral artery. Following coronary angiography or percutaneous coronary intervention, NOGA was conducted using a Smart-I Fiber (S8-6k, Amphi Co. Ltd., Osaka, Japan) and console (InterTec Medicals Co. Ltd., Osaka, Japan). Angioscopic images were captured using a digital video recorder for later offline analysis. Throughout the procedure, recordings were made while injecting 10 to 20 mL (1 - 2g) of dextran 40 and lactated Ringer's solution (Low Molecular Dextran L Injection; Otsuka Pharmaceutical Factory, Tokushima, Japan) to facilitate the removal of red blood cells from the observation site [12]. The total volume of solution administered during each examination ranged from approximately 50 mL to 80 mL, well below the threshold known to cause renal injury [13] [14]. Scanning of the aorta was conducted from the ascending to the terminal segments [6] [7] [15] [16] [17]. The tips of the fiber, 4-French probing catheter, and a 6-French Ikari Left 3.5 guiding catheter (Goodman, Nagoya, Japan) were aligned at a consistent position, and these catheters were then withdrawn from the ascending to the descending aorta while rotating to capture imaging of the entire aortic wall [18].

### 2.3 Assessment of non-obstructive general angioscopy

Aortic plaque refers to observable atherosclerosis affecting the inner surface of the aorta [19] [20]. Aortic plaque morphology was assessed based on previously published angioscopic descriptions, including those reported by Komatsu et al. [21]. Spontaneously ruptured aortic plaques (SRAPs) have been characterized by puff-like and/or chandelier-like appearances on NOGA, as described in the STROKE-NOGA study [22]. Plaque findings were classified into six morphological categories: puff chandelier rupture, puff rupture, thrombus, yellow plaque, ulcer, and fissure. The operational diagnostic criteria applied in the present study are summarized in Supplementary Table 1. Puff rupture was described as white or white-yellow puff-like materials easily dispersed spontaneously. Chandelier appearance was characterized by materials that glisten under the light from the tip of the fiber catheter during NOGA, without being blown out. Thrombi were characterized by red material adherent to the aortic intimal or plaque surface and protruding into the lumen, remaining visible during blood clearance. Yellow color grade (yellow grade) was defined as 0 (white), 1 (light yellow), 2 (yellow), or 3 (intense yellow) [23]. The presence of yellow plaques was defined as a maximum yellow color grade of 2 or 3 [24]. Ulcer and fissure were combined for quantitative analyses because both represent surface-disruption morphologies and because of their limited sample sizes (Supplementary Table 1). Operationally, puff chandelier rupture required both a dispersible white or white-yellow puff-like component and a glistening component that moved or wriggled under angioscopic illumination but was not washed out, consistent with published NOGA consensus standards [25]. In a previous NOGA study, Kojima et al. [16] reported excellent intraobserver (κ = 0.95) and interobserver (κ = 0.82) agreement for qualitative identification of aortic atheromatous plaques. Previous work by Yamaguchi et al. [26] also addressed angioscopic assessment of aortic plaque morphology. However, these previously reported findings do not directly establish the reproducibility of the six-category morphological classification applied in the present study. In the present study, when plaque morphology was difficult to classify, the recorded images were reviewed by two experienced investigators, and the final classification was determined by consensus. Formal interobserver agreement statistics for the present six-category classification were not prospectively obtained.

### 2.4 Detection of cholesterol crystals

CCs were detected using a previously reported filter paper rinse method [27]. A 10 mL blood sample was spread onto a 15 cm filter paper with a 7 μm pore size (no. 5A, Advantec Co., Ltd, Tokyo, Japan). The clear supernatant was then transferred onto a glass slide, covered with a glass plate, and sealed with resin [27]. The sample was examined under a polarized light microscope to confirm the presence of CCs (Fig. 1). Previous studies have demonstrated that aortic plaque is most abundant in the infrarenal abdominal aorta [9] [16], and 10 mL blood samples were collected via catheter from above the aortic plaque at each level of the second, third, and fourth lumbar vertebrae (L2, L3, and L4), which are considered representative sites for assessing systemic atherosclerosis. The present analysis included plaque-site samples with paired data on angioscopic morphology and CC assessment.

**Fig. 1:**
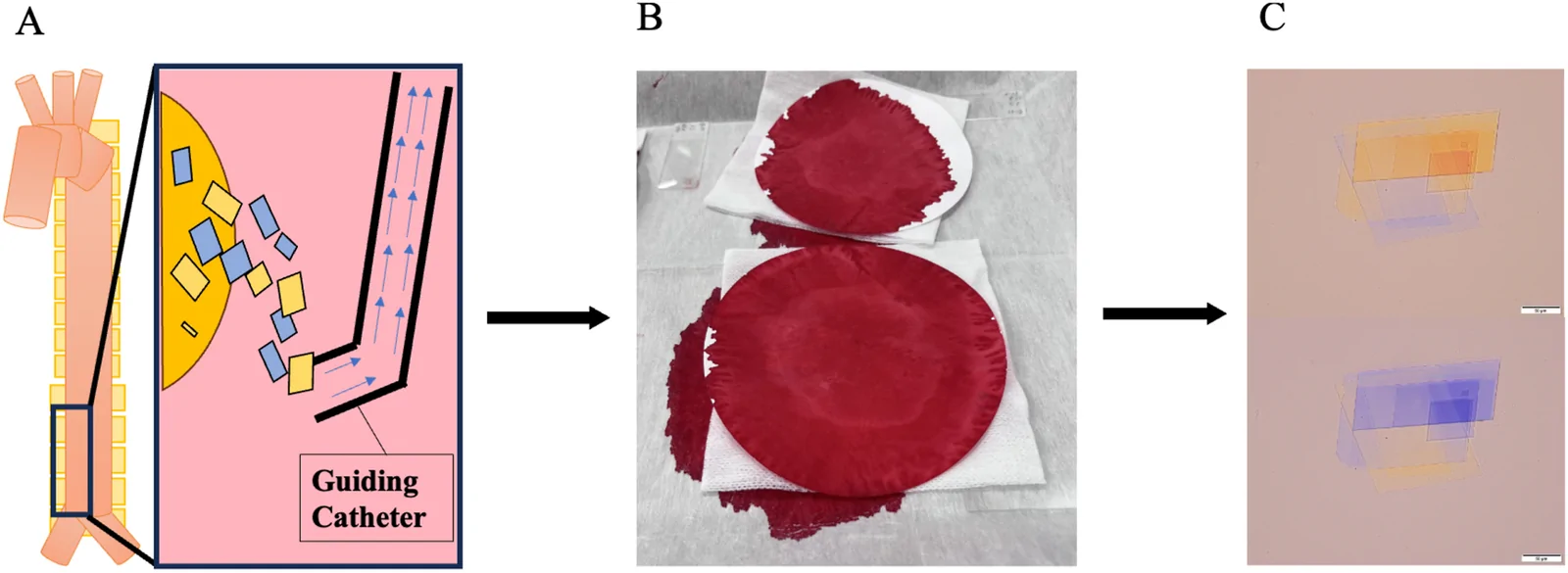
Cholesterol crystal detection. A: Ten-milliliter blood samples were collected via a catheter from above the aortic plaque at the level of the second, third, and fourth lumbar vertebrae. B: Blood samples were spread onto a 15-cm filter paper with a 7-μm pore size. C: The clear supernatant liquid was placed on a glass slide, covered with a glass plate, and sealed with resin. Samples were observed under a polarized light microscope to confirm the presence of cholesterol crystals.

### 2.5 Study endpoints

Aortic plaques exhibit various morphologies, but it remains unclear which plaque types are associated with CC detection in locally sampled blood. Therefore, the primary objective of this study was to determine whether specific NOGA-defined aortic plaque morphologies were associated with CC detection. The frequency of CC detection across plaque types was interpreted as an indirect marker of plaque activity and CC shedding potential.

### 2.6 Statistical analysis

Continuous variables were reported as mean with standard deviation for normally distributed data or median with interquartile range (IQR) for non-normally distributed data. Categorical variables were presented as counts and percentages. Comparisons of continuous variables were performed using Student's t-test for normally distributed data or Wilcoxon's rank sum test for non-normally distributed data. Categorical variables were presented as counts and percentages and analyzed using the chi-squared test or Fisher's exact test, as appropriate. For comparisons of CC detection rates among plaque morphologies, pairwise comparisons were performed using Fisher's exact test with puff chandelier rupture as the reference category. Because multiple plaque sites were obtained from the same patient, site-level associations between plaque morphology and CC detection were further evaluated using mixed-effects logistic regression with a binomial distribution and logit link, including a patient-specific random intercept to account for within-patient clustering. Plaque morphology was entered as a categorical fixed effect, with puff chandelier rupture as the reference category. Mixed-effects logistic regression was designated as the primary inferential analysis, whereas Fisher's exact test was used for exploratory comparisons of CC detection rates. The results of the mixed-effects logistic regression were expressed as odds ratios (ORs) with 95% confidence intervals (CIs) and unadjusted P values. Patient-level factors associated with CC detection were examined using univariate and multivariable logistic regression analyses. To limit model complexity and avoid overfitting, two clinically structured multivariable models were constructed without automated stepwise selection. Covariates were selected a priori based on their clinical relevance to atherosclerosis and CC detection rather than solely on statistical significance in univariate analyses. The number of puff chandelier ruptures, the principal angioscopic variable of interest, was included in both models. Model 1 included age, sex, history of myocardial infarction, history of cerebral infarction, history of peripheral artery disease, and the number of puff chandelier ruptures (n = 144). Model 2 included age, sex, LDL-C, HbA1c, eGFR, CRP, and the number of puff chandelier ruptures and was performed using complete-case analysis (n = 133). Correlated lipid variables were not entered simultaneously. Multicollinearity was assessed using variance inflation factors (VIFs); all VIFs were < 1.5 in both models (maximum VIF, 1.44 in Model 1 and 1.49 in Model 2), indicating no substantial multicollinearity. Model stability was further assessed by examining the consistency of the association between puff chandelier rupture burden and CC detection across the two independently specified adjustment models. The results of patient-level logistic regression analyses were presented as odds ratios (ORs) with 95% confidence intervals (CIs). All statistical analyses, including the multivariable logistic regression analysis, were performed using JMP Pro software version 16.1.0 (SAS Institute, Cary, USA). As an additional exploratory analysis, statin use and dose-based statin categories were compared between patients with and without a history of myocardial infarction. Categorical variables were compared using Fisher's exact test, and the overall distribution of statin dose categories was compared using the chi-square test. LDL-C levels were compared using the Wilcoxon rank-sum test. A p-value < 0.05 was considered statistically significant.

## 3 Results

### 3.1 Study patients

The baseline characteristics of the study population are summarized in Table 1**.** We analyzed 144 consecutive patients with CAD who underwent NOGA at our hospital between September 2021 and December 2024. Of these patients, 40.3% had diabetes mellitus, 97.2% had dyslipidemia, 76.4% had hypertension, 45.1% had a history of myocardial infarction, 20.8% had a history of peripheral artery disease, and 4.2% had a history of cerebral infarction. CCs were detected in 61 patients. Statins were prescribed in 98.6% of patients for secondary prevention. The mean level of low-density lipoprotein cholesterol (LDL-C) was 73 ± 36 mg/dL. Patients were divided into two groups based on the detection of CCs. There were no significant differences between the two groups in terms of age, sex, or coronary risk factors. In the CCs detected group, levels of total cholesterol (mean, 162 ± 46 mg/dL vs. 140 ± 36 mg/dL; p = 0.002), LDL-C (mean, 85 ± 43 mg/dL vs. 65 ± 27 mg/dL; p < 0.001), non-high-density lipoprotein cholesterol (non–HDL-C) (mean, 110 ± 43 mg/dL vs. 89 ± 36 mg/dL; p = 0.010) were significantly higher. In an additional analysis according to a history of myocardial infarction (MI), statin use was comparable between patients with and without prior MI (100% vs. 97.5%; P = 0.501). However, among statin-treated patients, the proportion receiving strong statin therapy, based on the dose categories defined in the present study, was significantly higher in those with prior MI (83.1% vs. 51.9%; P < 0.001). Conversely, moderate statin therapy was less frequent in patients with prior MI (10.8% vs. 37.7%; P < 0.001). The overall distribution of statin dose categories differed significantly between the groups (P < 0.001). In addition, patients with prior MI were more frequently treated with ezetimibe and/or PCSK9 inhibitors (69.2% vs. 36.7%; P < 0.001) and had lower LDL-C levels (62.0 vs. 69.5 mg/dL; P = 0.017) (Supplementary Table 2).

**Table 1:** Baseline characteristics and NOGA findings of patients divided into two groups according to CC detection.

Variables	Overall (n = 144)	CCs detected (n = 61)	Non-CCs detected (n = 83)	p value
Baseline characteristics				
Demographic variables				
Age, year	68 ± 10	69 ± 10	67 ± 10	0.15
Male, n, (%)	121 (84.0)	55 (90.2)	66 (79.5)	0.11
BMI, kg/m2	23.4 (21.3, 26.3)	23.2 (21.4, 26.3)	23.5 (21.1, 26.5)	0.78
Coronary risk factors				
Dyslipidemia, n, (%)	140 (97.2)	59 (96.7)	81 (97.6)	1.00
Diabetes mellitus, n, (%)	58 (40.3)	26 (42.6)	32 (38.6)	0.73
Hypertension, n, (%)	110 (76.4)	45 (73.8)	65 (78.3)	0.56
Smoking history, n, (%)	104 (72.2)	45 (73.8)	59 (71.1)	0.85
Medical history				
history of myocardial infarction, n, (%)	65 (45.1)	19 (31.1)	46 (55.4)	**0.004**
history of cerebral infarction, n, (%)	6 (4.2)	2 (3.3)	4 (4.8)	1.00
history of peripheral artery disease, n, (%)	30 (20.8)	19 (31.1)	11 (13.3)	**0.012**
Chronic kidney disease, n, (%)	55 (38.2)	23 (37.7)	32 (38.6)	1.00
Laboratory variables at admission				
Hemoglobin, g/dl	13.5 (12.4, 14.6)	13.6 (12.5, 14.7)	13.5 (12.2, 14.6)	0.71
Creatinine, mg/dl	1.27 ± 1.43	1.26 ± 1.45	1.27 ± 1.43	0.94
eGFR, ml/min/1.73 m2	60.5 ± 20.8	61.9 ± 22.6	59.5 ± 19.4	0.50
HbA1c, %	6.3 ± 0.8	6.3 ± 0.7	6.3 ± 0.9	0.81
Total cholesterol, mg/dl	149 ± 42	162 ± 46	140 ± 36	**0.002**
HDL-C, mg/dl	50 ± 15	48 ± 11	51 ± 17	0.16
LDL-C, mg/dl	73 ± 36	85 ± 43	65 ± 27	**< 0.001**
Triglyceride, mg/dl	127 ± 72	139 ± 84	118 ± 60	0.08
Non-HDL-C, mg/dl	98 ± 40	110 ± 43	89 ± 36	**0.010**
Uric acid, mg/dl	5.4 (4.3, 6.1)	5.3 (4.3, 6.1)	5.4 (4.4, 6.2)	0.44
CRP, mg/dl	0.28 ± 0.57	0.31 ± 0.71	0.25 ± 0.45	0.59
U alb/cre	23.5 ± 29.5	28.6 ± 34.3	12.1 ± 10.3	0.38
Medication				
Statin, n, (%)	142 (98.6)	60 (98.4)	82 (98.8)	1.00
Ezetimibe, n, (%)	72 (50.0)	26 (42.6)	46 (55.4)	0.18
PCSK9-I, n, (%)	5 (3.5)	2 (3.3)	3 (3.6)	1.00
Antiplatelet, n, (%)	129 (89.6)	53 (86.9)	76 (91.6)	0.42
Oral anticoagulant, n, (%)	16 (11.1)	5 (8.2)	11 (13.3)	0.43
Ras-I, n, (%)	117 (81.3)	44 (72.1)	73 (88.0)	**0.019**
Calcium channel blocker, n, (%)	61 (42.4)	31 (50.8)	30 (36.2)	0.09
Beta-blocker, n, (%)	95 (66.0)	39 (63.9)	56 (67.5)	0.72
SGLT2-I, n, (%)	61 (42.4)	26 (43.3)	35 (42.7)	1.00
NOGA findings per patient				
Presence				
Puff chandelier rupture, n, (%)	113 (78.4)	58 (95.1)	55 (66.2)	**< 0.001**
Puff rupture, n, (%)	89 (61.8)	40 (65.6)	49 (59.0)	0.49
Thrombi, n (%)	137 (95.1)	60 (98.4)	77 (92.8)	0.24
Yellow plaque, n, (%)	144 (100.0)	61 (100.0)	83 (100.0)	1.00
Ulcer + Fissure, n, (%)	95 (66.0)	43 (70.5)	52 (62.7)	0.38
Number				
Puff chandelier rupture, n	2 [1 - 4]	3 [2 - 6]	1 [0 - 3]	**< 0.001**
Puff rupture, n	1 [0 - 2]	1 [0 - 2]	1 [0 - 2]	0.66
Thrombi, n	4 [2 - 6]	4 [3 - 7]	3 [2 - 5]	**0.013**
Yellow plaque, n	12 [8−14]	12 [10 - 15]	11 [7−14]	**0.032**
Ulcer + Fissure, n	2 [0 - 3]	2 [1 -3]	2 [0 - 3]	0.29

Significant p values are written in bold.

Data are expressed as mean ± SD, median (interquartile range), or n (%).

NOGA, non-obstructive general angioscopy; CCs, cholesterol crystals; Non-CCs detected, patients without detected cholesterol crystals; BMI, body mass index; eGFR, estimated glomerular filtration rate; HbA1c, Hemoglobin A1c; HDL-C, high-density lipoprotein cholesterol; LDL-C, low-density lipoprotein cholesterol; Non-HDL-C, Non-high density lipoprotein cholesterol; CRP, C-reactive protein; U alb/cre, urinary albumin-to-creatinine ratio; PCSK9-I, proprotein convertase subtilisin kexin 9 inhibitor; Ras-I, renin-angiotensin system inhibitor; SGLT2-i, sodium-glucose cotransporter 2 inhibitor.

### 3.2 NOGA findings

Aortic plaques were assessed at 350 vertebral-level observation sites in the infrarenal abdominal aorta using NOGA and classified into six morphological categories (Fig. 2). Six plaque morphologies were identified; ulcer and fissure were combined for quantitative analyses. Blood samples were collected from representative plaques at L2, L3, and L4 vertebral levels. Among the sampling sites, puff chandelier rupture accounted for 46.3%, puff rupture for 12.3%, thrombi for 9.7%, yellow plaque for 18.6%, and ulcer and fissure for 13.1% (Fig. 3). Table 1 summarizes the NOGA findings in the aorta per patient stratified by the presence or absence of CCs. In the CCs group, puff chandelier rupture was more frequently observed (58 [95.1%] vs. 55 [66.2%]; p < 0.001). Furthermore, the number of puff chandelier ruptures was significantly higher (median, 3.0 [IQR: 2.0-6.0] vs. 1.0 [IQR: 0.0-3.0]; p < 0.001), as were the number of thrombi (median, 4.0 [IQR: 3.0-7.0] vs. 3.0 [IQR: 2.0-5.0]; p = 0.013) and the number of yellow plaques was higher (median, 12.0 [IQR: 10.0-15.0] vs. 11.0 [IQR: 7.0-14.0]; p = 0.032).

**Fig. 2:**
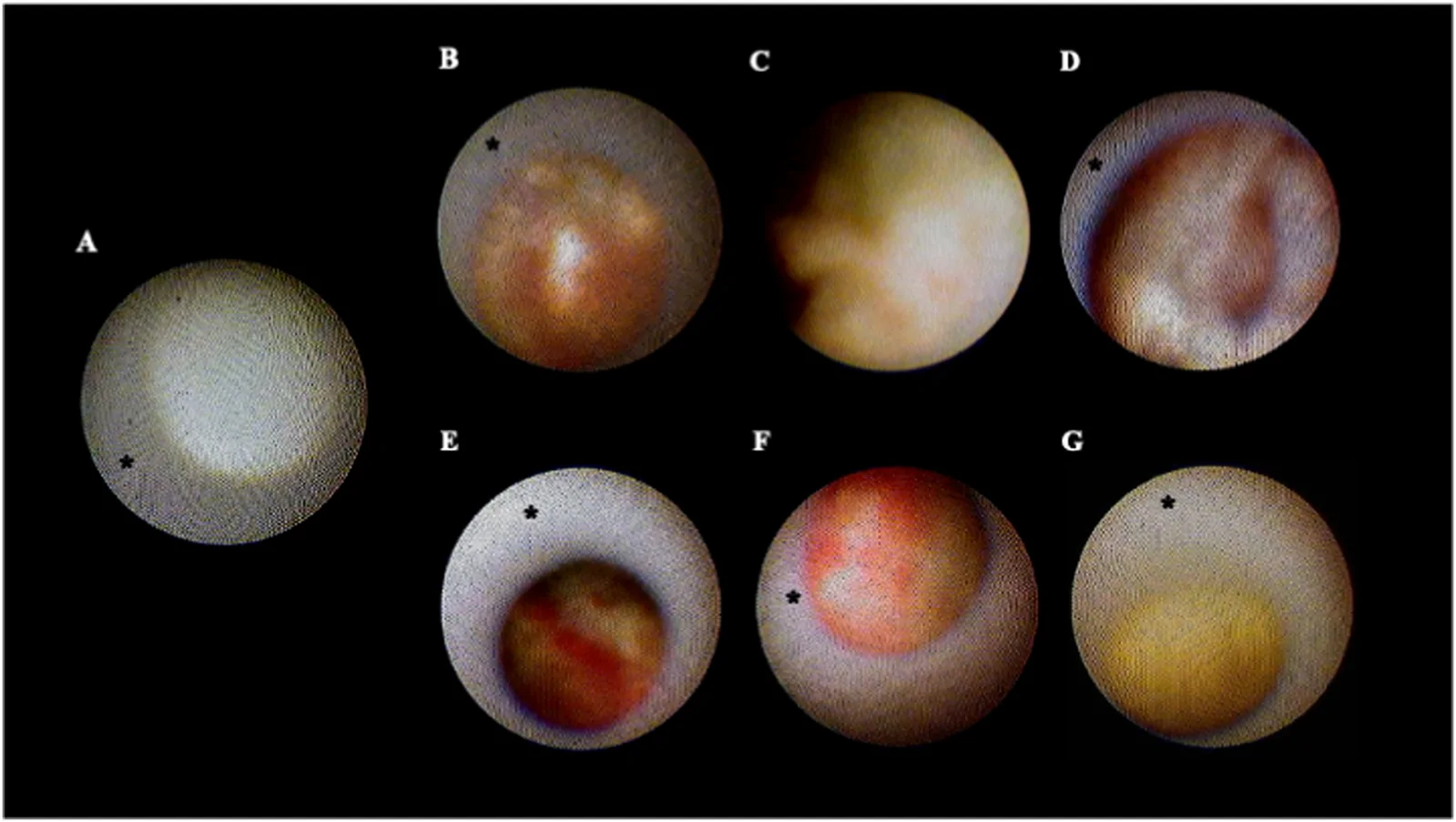
Representative angioscopic findings of aortic plaque morphologies. A: Normal intima, B: Puff chandelier rupture, C: Puff rupture, D: Ulcer, E: Fissure, F: Thrombi, G: Yellow plaque. The asterisk indicates the probing catheter.

**Fig. 3:**
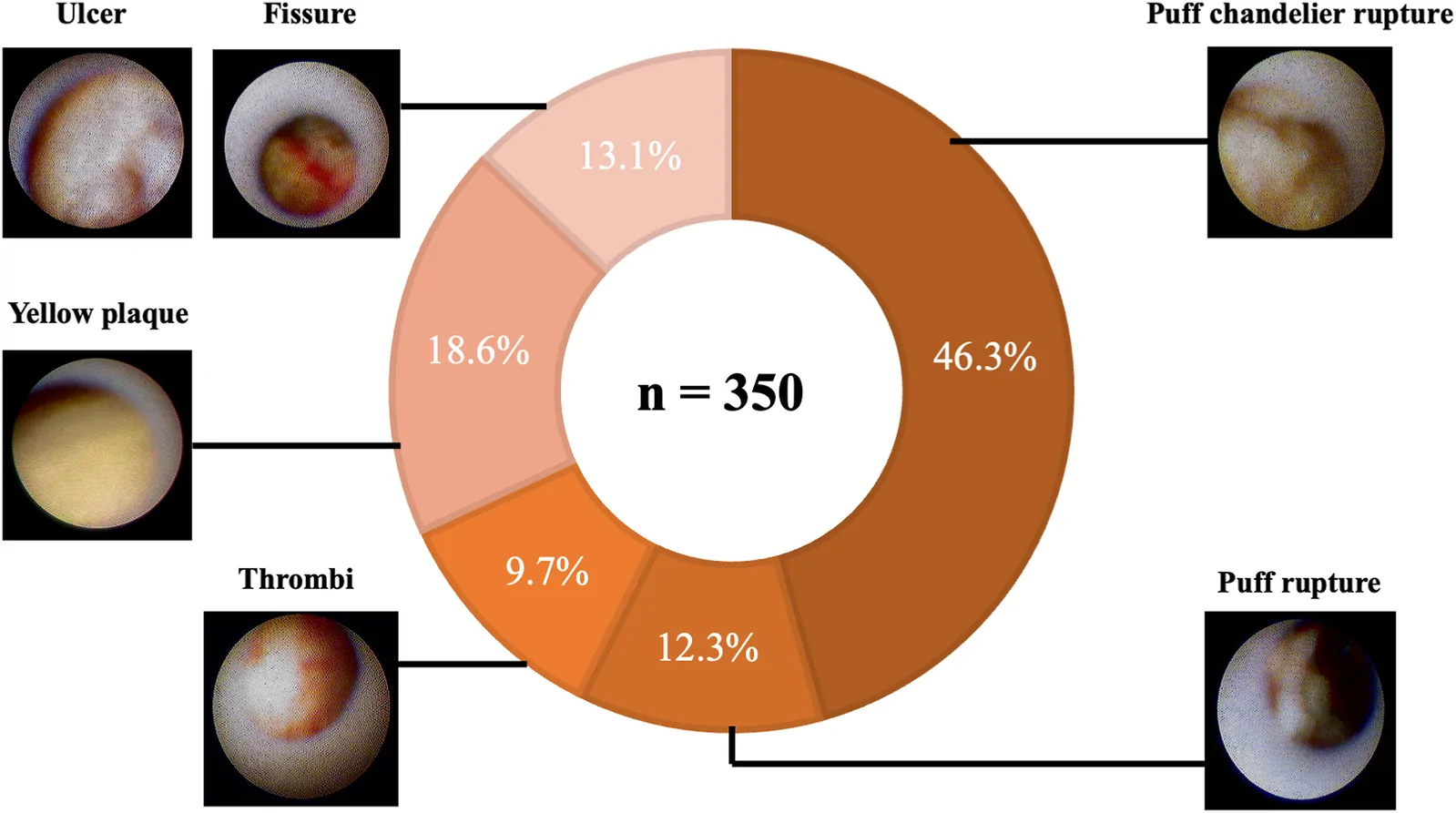
Frequency of aortic plaque characteristics observed by NOGA at the sampling site Aortic plaques at 350 locations in the infrarenal abdominal aorta were observed and classified into six morphological characteristics. Blood samples were obtained from representative plaques at each level of the second, third, and fourth lumbar vertebrae. Puff chandelier ruptures accounted for 46.3% of the sampling sites, puff ruptures for 12.3%, thrombi for 9.7%, yellow plaques for 18.6%, and ulcers and fissures for 13.1%. NOGA, non-obstructive general angioscopy.

### 3.3 Association between types of aortic plaque and detection rate of cholesterol crystals

CCs were detected at 79 of 350 plaque sites (22.6%). The detection rate of CCs was highest in puff chandelier ruptures at 42.6% (69/162), followed by puff ruptures at 16.3% (7/43), thrombi at 2.9% (1/34), ulcers and fissures at 2.2% (1/46), and yellow plaques at 1.5% (1/65). Ulcers and fissures were analyzed together in the primary site-level analysis because of the small number of events. When reported separately, CCs were detected in 1 of 33 ulcer sites (3.0%) and in 0 of 13 fissure sites (0%) (Fig. 4). After accounting for within-patient clustering using a mixed-effects logistic regression model with a patient-specific random intercept, plaque morphology remained significantly associated with CC detection (global P < 0.0001). Compared with puff chandelier rupture, the odds of CC detection were significantly lower for all other plaque morphologies (Supplementary Table 3). Fig. 5 illustrates a representative case. At the L2 vertebral level, the plaque was classified as a puff rupture, and no CCs were detected. At the L3 level, the plaque was a thrombus; again, no CCs were detected. At the L4 level, a puff chandelier rupture was observed, and CCs were detected in the sample obtained from this level.

**Fig. 4:**
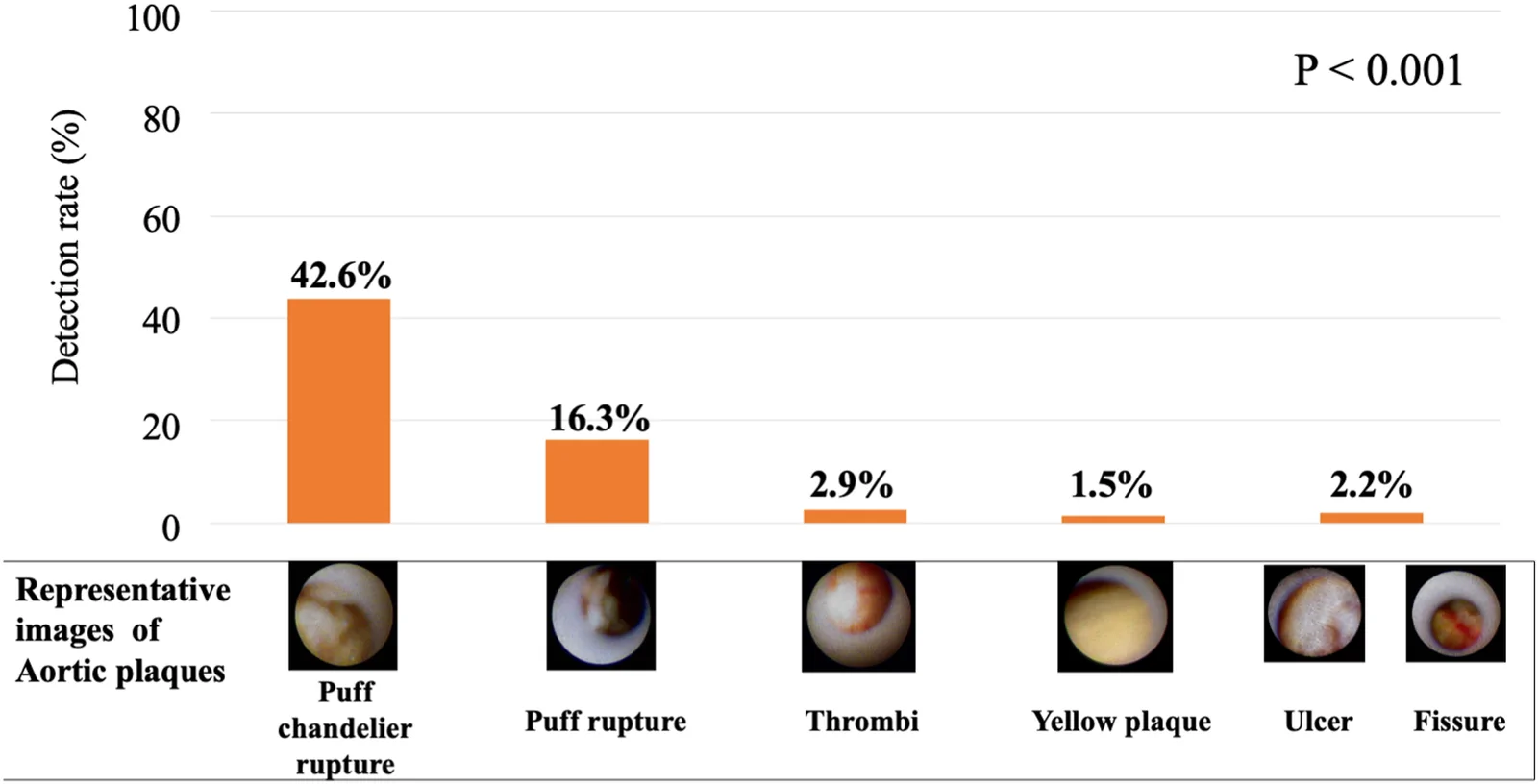
Detection rate of cholesterol crystals according to aortic plaque morphology Cholesterol crystals were detected in 79 of 350 aortic plaques (22.6%). The detection rate was 42.6% (69/162) for puff chandelier ruptures, 16.3% (7/43) for puff ruptures, 2.9% (1/34) for thrombi, 1.5% (1/65) for yellow plaques, and 2.2% (1/46) for ulcers and fissures. The global association between plaque morphology and CC detection was assessed using mixed-effects logistic regression with a patient-specific random intercept (P < 0.0001). Pairwise comparisons are presented in Supplementary Table 3.

**Fig. 5:**
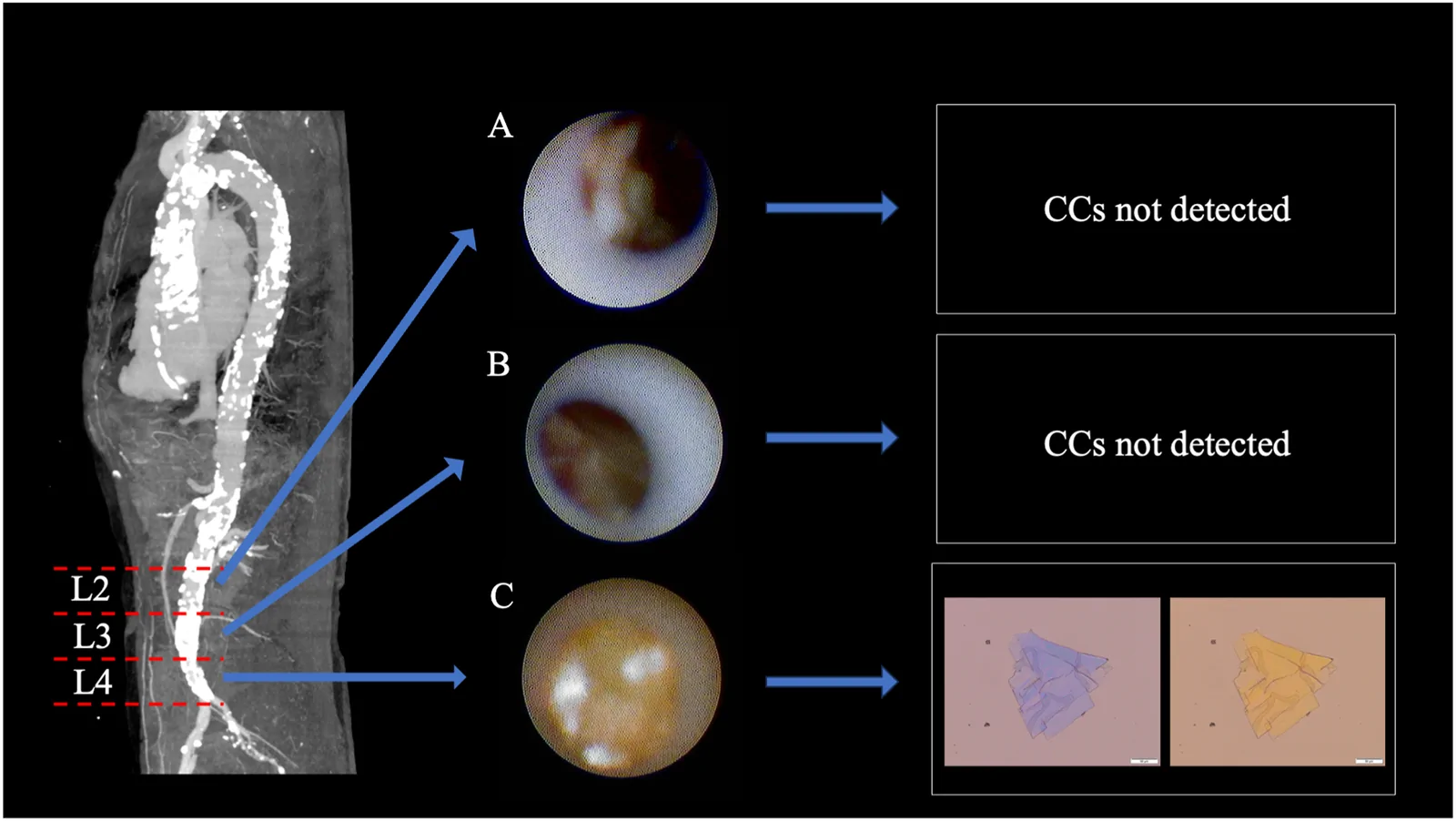
Representative aortic plaque morphologies assessed by non-obstructive general angioscopy and cholesterol crystal detection in locally sampled blood. A: The plaque at the level of the second lumbar vertebra (L2) was a puff rupture, and cholesterol crystals were not detected. B: The plaque at the level of the third lumbar vertebra (L3) was a thrombus, and cholesterol crystals were not detected. C: The plaque at the level of the fourth lumbar vertebra (L4) was a puff chandelier rupture, and cholesterol crystals were detected.

### 3.4 Univariate and multivariable analyses to identify factors associated with cholesterol crystals

In the univariate analysis, several clinical, laboratory, medication, and angioscopic variables were significantly associated with CC detection, including a history of myocardial infarction, a history of peripheral artery disease, lipid parameters (total cholesterol, LDL-C, and non–HDL-C), use of RAS inhibitors, and angioscopic plaque findings (Table 2). Multivariable logistic regression analyses were performed using two different adjustment models, as shown in Table 2. In Model 1, which was adjusted for demographic variables and atherosclerotic comorbidities, the number of puff chandelier ruptures was independently associated with the presence of CCs (odds ratio [OR] 1.44; 95% confidence interval [CI] 1.19–1.75; p < 0.001). In contrast, a history of myocardial infarction showed a significant inverse association with CC presence (OR 0.43; 95% CI 0.19–0.95; p = 0.036). Other variables, including age, sex, history of cerebral infarction, and history of peripheral artery disease, were not significantly associated with CC presence (Table 2). In Model 2, which was adjusted for laboratory parameters, LDL-C was significantly associated with the presence of CCs (OR 1.02; 95% CI 1.01–1.03; p = 0.009). The number of puff chandelier ruptures remained independently associated with CC presence (OR 1.49; 95% CI 1.23–1.86; p < 0.001). No significant associations were observed for age, sex, HbA1c, eGFR, or CRP (Table 2).

**Table 2:** Univariate and multivariable logistic regression analyses of factors associated with cholesterol crystals.

Variables	Univariate analysis		
	Odds ratio	95% CI	p value
Demographic variables			
Age, year	1.03	0.99-1.06	0.15
Male, n	2.36	0.87-6.40	0.09
BMI, kg/m2	0.99	0.90-1.08	0.78
Coronary risk factors			
Dyslipidemia, n, (%)	0.72	0.10-5.32	0.76
Diabetes mellitus, n, (%)	1.18	0.60-2.32	0.62
Hypertension, n, (%)	0.78	0.36-1.69	0.53
Smoking history, n, (%)	1.14	0.55-2.40	0.72
Medical history			
history of myocardial infarction, n, (%)	0.36	0.18-0.73	**0.004**
history of cerebral infarction, n, (%)	0.67	0.19-3.78	0.65
history of peripheral artery disease, n, (%)	2.96	1.29-6.82	**0.011**
Chronic kidney disease, n, (%)	0.97	0.49-1.91	0.92
Laboratory variables at admission			
Hemoglobin, g/dl	1.02	0.82-1.27	0.85
Creatinine, mg/dl	0.99	0.79-1.25	0.94
eGFR, ml/min/1.73 m2	1.01	0.99-1.02	0.50
HbA1c, %	0.95	0.62-1.45	0.81
Total cholesterol, mg/dl	1.01	1.01-1.02	**0.003**
HDL-C, mg/dl	0.98	0.96-1.01	0.16
LDL-C, mg/dl	1.02	1.01-1.03	**0.002**
Triglyceride, mg/dl	1.00	1.00-1.01	0.09
Non-HDL, mg/dl	1.02	1.00-1.03	**0.016**
Uric acid, mg/dl	0.92	0.71-1.18	0.50
CRP, mg/dl	1.17	0.64-2.20	0.59
U alb/cre	1.03	0.96-1.11	0.39
Medication			
Statin, n, (%)	0.73	0.05-11.93	0.83
Ezetimibe, n, (%)	0.60	0.31-1.16	0.13
PCSK9-I, n, (%)	0.90	0.15-5.58	0.91
Antiplatelet, n, (%)	0.61	0.21-1.78	0.37
Oral anticoagulant, n, (%)	0.58	0.19-1.78	0.34
Ras-I, n, (%)	0.35	0.15-0.84	**0.019**
Calcium channel blocker, n, (%)	1.83	0.93-3.58	0.08
Beta-blocker, n, (%)	0.86	0.43-1.71	0.66
SGLT2-I, n, (%)	1.03	0.52-2.01	0.94
Presence of aortic plaques			
Puff chandelier rupture, n, (%)	9.84	2.83-34.23	**< 0.001**
Puff rupture, n, (%)	1.32	0.67-2.62	0.43
Thrombi, n, (%)	4.67	0.54-39.89	0.16
Ulcer + Fissure, n, (%)	1.42	0.70-2.89	0.33
Number of aortic plaques			
Puff chandelier rupture, n	1.51	1.27-1.79	**< 0.001**
Puff rupture, n	1.07	0.85-1.35	0.58
Thrombi, n	1.21	1.06-1.38	**0.005**
Yellow plaque, n	1.09	1.00-1.18	**0.045**
Ulcer + Fissure, n	1.07	0.89-1.28	0.49

Variables	Multivariate analysis		
	Odds ratio	95% CI	p value
Model 1			
Age	1.01	0.96-1.04	0.98
Sex (Male)	2.75	0.87-9.15	0.09
History of MI	0.43	0.19-0.95	**0.036**
History of CI	0.43	0.05-3.48	0.42
History of PAD	1.61	0.56-4.60	0.38
Number of puff chandelier ruptures	1.44	1.19-1.75	**< 0.001**
Model 2			
Age	1.03	0.98-1.09	0.26
Sex (Male)	2.51	0.68-9.20	0.16
LDL-C	1.02	1.01-1.03	**0.009**
HbA1c	1.02	0.59-1.72	0.94
eGFR	1.02	1.00-1.05	0.07
CRP	1.26	0.62-2.70	0.53
Number of puff chandelier ruptures	1.49	1.23-1.86	**< 0.001**

Model 1; Age, Sex, history of myocardial infarction, history of cerebral infarction, history of peripheral artery disease, number of puff chandelier rupture

Model 2; Age, Sex, LDL-C, HbA1c, eGFR, CRP, number of puff chandelier rupture.

CI, confidence interval; BMI, body mass index; eGFR, estimated glomerular filtration rate; HbA1c, Hemoglobin A1c; HDL-C, high-density lipoprotein cholesterol; LDL-C, low-density lipoprotein cholesterol; Non-HDL, Non-high density lipoprotein cholesterol; CRP, C-reactive protein; U alb/cre, urinary albumin-to-creatinine ratio; PCSK9-I, proprotein convertase subtilisin kexin 9 inhibitor; Ras-I, renin-angiotensin system inhibitor; SGLT2-i, sodium-glucose cotransporter 2 inhibitor; MI, myocardial infarction; CI, cerebral infarction; PAD, peripheral artery disease.

## 4 Discussion

This study is the first, to our knowledge, to demonstrate a systematic and quantitative association between puff chandelier rupture morphology assessed by non-obstructive general angioscopy (NOGA) and the detection of cholesterol crystals (CCs) in locally sampled blood. Puff chandelier rupture showed the highest site-level CC detection rate among the evaluated plaque morphologies, and its burden remained independently associated with CC detection in patient-level multivariable analyses**.** Our previous EAST-NOGA study demonstrated that the overall burden of NOGA-detected aortic plaques was associated with subsequent vascular events [9], but it did not evaluate the biological activity associated with individual plaque morphologies. The present study extends these observations by directly linking a specific macroscopic plaque phenotype—puff chandelier rupture—with CC detection in locally sampled blood as an in vivo functional marker related to plaque activity and CC shedding potential in a consecutive cohort. Thus, the principal incremental value of the present study is the establishment of a morphology–function relationship. Previous clinical studies have also demonstrated the potential embolic significance of spontaneously ruptured aortic plaques (SRAPs). In particular, the STROKE-NOGA study reported a significant association between SRAPs, characterized by puff-like and/or chandelier-like appearances, and ischemic cerebral infarction [22]. The present study extends these observations by quantitatively evaluating differences in local cholesterol crystal (CC) detection among different aortic plaque morphologies. Notably, puff chandelier rupture (PCR) exhibited the highest CC detection rate, suggesting that distinct morphological patterns may reflect differences in biological activity. These findings provide further pathophysiological insight into the potential role of aortic plaques as sources of cholesterol crystal embolization. However, whether PCR independently predicts subsequent clinical embolic events remains to be determined.

Our angioscopic analysis revealed that puff chandelier rupture was the predominant plaque morphology in the infrarenal abdominal aorta. Together with puff ruptures and ulcerative lesions, these findings resemble complicated coronary plaques classified as type VI according to the American Heart Association [28]. In contrast to coronary arteries, however, the large caliber of the aorta renders thrombotic occlusion uncommon [21]; instead, aortic plaques may function as sources of CC release into the circulation [20] [29]. A previous case report documented branch retinal artery occlusion in a patient with a puff chandelier rupture in the aortic arch accompanied by CC release [30], suggesting a potential link between this morphology, CC shedding and distal embolization. However, such case-based evidence cannot establish clinical risk. Beyond case-based observations, a multicenter cohort of 167 patients undergoing percutaneous coronary intervention showed that the presence of puff chandelier rupture was independently associated with subsequent major adverse cardiac events (hazard ratio 3.73, 95% confidence interval 1.57-8.87), supporting the feasibility and clinical relevance of applying this morphology across centers [26]. The independent association between the number of puff chandelier ruptures and CC detection after adjustment for atherosclerotic comorbidities, lipid parameters, renal function, and systemic inflammation suggests that this morphology captures plaque characteristics related to CC detection beyond overall atherosclerotic burden.

The inverse association between a history of myocardial infarction and CC detection may partly reflect differences in secondary preventive lipid-lowering management. Although statin use was nearly universal in both groups, patients with prior MI were more frequently prescribed higher-dose statin regimens, as reflected by the greater proportion classified as receiving strong statin therapy. In addition, adjunctive treatment with ezetimibe and/or PCSK9 inhibitors was more common, and LDL-C levels were lower in patients with prior MI. These findings suggest that differences in overall lipid-lowering management may partly contribute to the observed association. However, the observational design does not establish a causal relationship, and residual confounding cannot be excluded. The association between LDL-C and CC detection is biologically plausible because lipid accumulation is closely linked to cholesterol crystallization and atherosclerotic plaque disruption [31] [32]. CCs themselves may further contribute to atherosclerotic inflammation through mechanisms, including NLRP3 inflammasome activation [20] [33] [34]. Previous serial NOGA observations have suggested that intensive lipid-lowering therapy may modify aortic plaque morphology [35] [36]; however, whether such treatment reduces CC detection or CC shedding remains to be determined in prospective studies.

Overall, the present findings identify puff chandelier rupture as a distinct aortic plaque morphology associated with a higher likelihood of cholesterol crystal detection in locally sampled blood, providing further pathophysiological insight into the potential embolic significance of aortic plaques. NOGA enables real-time, in vivo visualization of aortic plaque morphology and may provide information beyond that obtained from conventional imaging. Although NOGA is invasive and unsuitable for routine screening, it provides direct visualization of subtle aortic plaque surface disruptions and dynamic morphological features that may not be adequately assessed by conventional imaging modalities. In our previous study comparing multidetector computed tomography (CT) with NOGA, plaque ruptures were identified by NOGA even in aortic segments that appeared nearly normal on CT [5]. These findings highlight the complementary role of NOGA in characterizing aortic plaque morphology beyond conventional CT assessment.

The present study extends these observations by demonstrating a quantitative association between NOGA-defined plaque morphologies and cholesterol crystal detection in locally sampled blood. Although advances in non-invasive imaging may eventually allow more detailed assessment of these morphological features, the ability of current non-invasive modalities to reliably identify puff chandelier rupture or assess local cholesterol crystal release has not been established. Thus, the principal significance of our findings lies in linking directly observed aortic plaque morphology with local cholesterol crystal detection rather than demonstrating immediate applicability to non-invasive imaging.

## 5 Limitations

This study has several limitations. First, it was a single-center study with a relatively small sample size, which may limit generalizability. Second, because of its observational and cross-sectional design, causal relationships between plaque morphology and presence of CCs or release of CCs cannot be inferred. Third, although NOGA enables high-resolution in vivo plaque assessment, it is invasive and not applicable to all patients in routine practice. In addition, CC detection relied on blood samples obtained from specific infrarenal aortic segments, which may not fully represent the systemic atherosclerotic burden or overall plaque activity within the aorta, and the precise origin of CCs, including possible upstream sources, remains uncertain. Furthermore, plaque morphology was assessed at a single time point, precluding evaluation of the temporal dynamics of CC release. Finally, associations between CC detection and clinical characteristics, such as lipid levels or RAS-I use, should be interpreted cautiously due to limited sample size and residual confounding. Larger, multicenter studies with longitudinal follow-up are warranted to further validate these findings and clarify the relationship between plaque morphology, CC release, and clinical outcomes.

## 6 Conclusions

Aortic plaques classified as puff chandelier ruptures were associated with a significantly higher frequency of cholesterol crystal detection in locally sampled blood than other plaque morphologies. These findings provide further pathophysiological insight into the potential embolic significance of spontaneously ruptured aortic plaques. However, further longitudinal studies are needed to determine whether puff chandelier rupture independently predicts clinical embolic events.

## Ethics declaration

The authors have NOT obtained informed consent from participants or their legal representatives. The requirement for individual informed consent was waived by the Institutional Review Board of Nihon University Itabashi Hospital (approval number: RK-261013-14). This observational study was conducted using an opt-out consent procedure approved by the Institutional Review Board. Information regarding the study was made publicly available, and patients were provided with an opportunity to decline participation.

This study involved human biological materials, specifically blood samples collected from patients. The use of these samples for the present observational study was approved by the Institutional Review Board of Nihon University Itabashi Hospital (approval number: RK-261013-14). The requirement for individual informed consent was waived by the Institutional Review Board, and the study was conducted using an approved opt-out procedure. Information about the study was made publicly available, and patients were provided with an opportunity to decline participation.

This study was performed in compliance with relevant laws, regulatory frameworks and guidelines where the research took place. This study was conducted in accordance with Declaration of Helsinki and Ethical Guidelines for Medical and Biological Research Involving Human Subjects in Japan. This study was approved by the Institutional Review Board of Nihon University Itabashi Hospital. (Approval No. RK-261013-14)

## Ethics approval and consent to participate

The study was approved by the Institutional Review Board of Nihon University Itabashi Hospital (RK-261013-14) and was registered in the University Hospital Medical Information Network Clinical Trials Registry of Japan (UMIN000051008). The requirement for individual informed consent was waived by the Institutional Review Board, and the study was conduced using an approved opt-out consent procedure. Information regarding the study was made publicity available, and patients were provided with an opportunity to decline participation.

## Data availability

The data, analytic methods, and study materials will be made available from the corresponding author on reasonable request for purposes of reproducing the results.

## Funding

This work was supported by JSPS KAKENHI Grant Number 21K16041 and the 10.13039/100029577Japan Arteriosclerosis Prevention Fund.

## Declaration of competing interests

The authors declare that they have no known competing financial interests or personal relationships that could have appeared to influence the work reported in this paper.

## Appendix A Supplementary data

Multimedia component 3

Supplementary Video 1 Puff chandelier rupture.

Supplementary Video 2 Puff rupture.

Supplementary Video 3 Ulcer.

Supplementary Video 4 Fissure.

Supplementary Video 5 Thrombi.

Supplementary Video 6 Yellow plaque.

## References

[1] Soehnlein O., Lutgens E., Doring Y. (2025). Distinct inflammatory pathways shape atherosclerosis in different vascular beds. Eur Heart J.

[2] Kronzon I., Tunick P.A. (2006). Aortic atherosclerotic disease and stroke. Circulation.

[3] Amarenco P. (1994). Atherosclerotic disease of the aortic arch and the risk of ischemic stroke. N Engl J Med.

[4] Hess C.N. (2019). Acute limb ischemia in peripheral artery disease. Circulation.

[5] Miyagawa M. (2024). Association between aortic wall parameters on multidetector computed tomography and ruptured plaques by nonobstructive general angioscopy. J Am Heart Assoc.

[6] Komatsu S. (2015). Early detection of vulnerable atherosclerotic plaque for risk reduction of acute aortic rupture and thromboemboli and atheroemboli using non-obstructive angioscopy. Circ J.

[7] Aono J. (2015). Correlation between plaque vulnerability of aorta and coronary artery: an evaluation of plaque activity by direct visualization with angioscopy. Int J Cardiovasc Imag.

[8] Mizobuchi S. (2024). Association between aortic thrombi detected using non-obstructive general angioscopy and atrial fibrillation. J Thromb Thrombolysis.

[9] Kojima K. (2022). Aortic plaque burden predicts vascular events in patients with cardiovascular disease: the EAST-NOGA study. J Cardiol.

[10] Virmani R. (2006). Pathology of the vulnerable plaque. J Am Coll Cardiol.

[11] Jia H. (2013). In vivo diagnosis of plaque erosion and calcified nodule in patients with acute coronary syndrome by intravascular optical coherence tomography. J Am Coll Cardiol.

[12] Komatsu S. (2017). Improving the visual field in coronary artery by with non-obstructive angioscopy: dual infusion method. Int J Cardiovasc Imag.

[13] Feest T.G. (1976). Low molecular weight dextran: a continuing cause of acute renal failure. Br Med J.

[14] Biesenbach G., Kaiser W., Zazgornik J. (1997). Incidence of acute oligoanuric renal failure in dextran 40 treated patients with acute ischemic stroke stage III or IV. Ren Fail.

[15] Komatsu S. (2018). Subintimal intraplaque haemorrhage prior to aortic plaque rupture: 2-year angioscopic follow-up. BMJ Case Rep.

[16] Kojima K. (2019). Aortic plaque distribution, and association between aortic plaque and atherosclerotic risk factors: an aortic angioscopy study. J Atheroscler Thromb.

[17] Nishi H. (2020). Aortic angioscopy assisted thoracic endovascular repair for chronic type B aortic dissection. J Cardiol.

[18] Kojima K. (2021). High wall shear stress is related to atherosclerotic plaque rupture in the aortic arch of patients with cardiovascular disease: a study with computational fluid dynamics model and non-obstructive general angioscopy. J Atheroscler Thromb.

[19] Komatsu S. (2018). Angioscopic evaluation of spontaneously ruptured aortic plaques. J Am Coll Cardiol.

[20] Komatsu S. (2022). Debris collected in-situ from spontaneously ruptured atherosclerotic plaque invariably contains large cholesterol crystals and evidence of activation of innate inflammation: insights from non-obstructive general angioscopy. Atherosclerosis.

[21] Komatsu S. (2020). Spontaneous ruptured aortic plaque and injuries: insights for aging and acute aortic syndrome from non-obstructive general angioscopy. J Cardiol.

[22] Higuchi Y. (2022). Significant contribution of aortogenic mechanism in ischemic stroke: observation of aortic plaque rupture by angioscopy. JACC Asia.

[23] Ueda Y. (1996). Intracoronary morphology of culprit lesions after reperfusion in acute myocardial infarction: serial angioscopic observations. J Am Coll Cardiol.

[24] Ueda Y. (2015). In-Stent yellow plaque at 1 year after implantation is associated with future event of very late stent failure: the DESNOTE study (Detect the event of very late stent failure from the drug-eluting stent not well covered by Neointima determined by angioscopy). JACC Cardiovasc Interv.

[25] Hiro T. (2018). Consensus standards for acquisition, measurement, and reporting of non-obstructive aortic angioscopy studies: a report from the working group of Japan vascular imaging research organization for standardization of non-obstructive aortic angioscopy (Version 2017). Angioscopy.

[26] Yamaguchi M. (2020). Clinical significance of the presence of puff-chandelier ruptures detected by nonobstructive aortic angioscopy. Catheter Cardiovasc Interv.

[27] Iwa N. (2022). Novel methods for detecting human cholesterol crystals from sampled blood. Lab Med.

[28] Stary H.C. (1995). A definition of advanced types of atherosclerotic lesions and a histological classification of atherosclerosis. A report from the committee on vascular lesions of the council on arteriosclerosis, American Heart Association. Circulation.

[29] Writing Committee M. (2022). ACC/AHA guideline for the diagnosis and management of aortic disease: a report of the American heart association/American college of cardiology joint committee on clinical practice guidelines. J Am Coll Cardiol.

[30] Migita S., Kojima K., Okumura Y. (2024). Branch retinal artery occlusion with non-obstructive general angioscopy confirmed puff-chandelier rupture releasing cholesterol crystals in the aortic arch: a case report. Eur Heart J Case Rep.

[31] Komatsu S. (2023). Cholesterol crystals as the main trigger of Interleukin-6 production through innate inflammatory response in human spontaneously ruptured aortic plaques. J Atheroscler Thromb.

[32] Komatsu S. (2024). Different characteristics and Interleukin-6 ratios of scattering-type aortic plaques. Cureus.

[33] Duewell P. (2010). NLRP3 inflammasomes are required for atherogenesis and activated by cholesterol crystals. Nature.

[34] Abela G.S. (2009). Effect of cholesterol crystals on plaques and intima in arteries of patients with acute coronary and cerebrovascular syndromes. Am J Cardiol.

[35] Kojima K., Ebuchi Y., Okumura Y. (2025). Efficacy of aggressive lipid-lowering therapy on systemic plaque stabilisation in acute myocardial infarction, assessed using non-obstructive general angioscopy. Eur Heart J Case Rep.

[36] Ebuchi Y. (2022). Serial changes of aortic vulnerable plaques observed via non-obstructive general angioscopy. Int Heart J.

