# Supplementary material for: Aortic plaque morphology and cholesterol crystal release assessed using non-obstructive general angioscopy
Source: Atheroscler Plus. 2026 Nov 7;66:100623. doi: 10.1016/j.athplu.2026.100623 (PMC13770710; doi:10.1016/j.athplu.2026.100623)
Supplement: Supplementary file 1 [file mmc1.docx]

Supplementary Table 1. Operational diagnostic criteria for aortic plaque morphology assessed by NOGA

| Aortic plaque morphology | Diagnostic criteria on NOGA | | | | | | | |  |
| --- | --- | --- | --- | --- | --- | --- | --- | --- | --- |
| Puff chandelier rupture | Presence of both **puff rupture and chandelier appearance**: white or white-yellow puff-like material that is spontaneously dispersed, together with material that glistens under illumination from the angioscopic fiber and remains mobile without being dispersed. | | | | | | | |  |
|  |  |  |  |  |  |  |  |  |  |
| Puff rupture | **White or white-yellow puff-like material** arising from the plaque surface that is **easily and spontaneously dispersed by blood flow**. | | | | | | | |  |
|  |  |  |  |  |  |  |  |  |  |
| Thrombi | **Red thrombotic material** adhering to the aortic intimal or plaque surface, resembling a strawberry-jam appearance, and not washed away by low-molecular-weight dextran infusion. | | | | | | | |  |
|  |  |  |  |  |  |  |  |  |  |
| Yellow plaque | Plaque with a **maximum yellow color grade of 2 or 3** on the 4-grade angioscopic scale: grade 0, white; grade 1, light yellow; grade 2, yellow; and grade 3, intense yellow. | | | | | | | |  |
|  |  |  |  |  |  |  |  |  |  |
| Ulcer | A localized ulcerative depression or defect of the plaque surface, deeper than an erosion, indicating disruption of the aortic intimal surface. | | | | | | | |  |
|  |  |  |  |  |  |  |  |  |  |
| Fissure | An **elongated cleft or tear** in the plaque or aortic intimal surface. | | | | | | | |  |
|  |  |  |  |  |  |  |  |  |  |

The morphological criteria were based on previously published angioscopic descriptions, including those reported by Komatsu et al. [21], and adapted for the present study. Yellow plaque was defined according to previously established angioscopic color grading criteria [23.24].

NOGA, non-obstructive general angioscopy.
