# Supplementary material for: Aortic plaque morphology and cholesterol crystal release assessed using non-obstructive general angioscopy
Source: Atheroscler Plus. 2026 Nov 7;66:100623. doi: 10.1016/j.athplu.2026.100623 (PMC13770710; doi:10.1016/j.athplu.2026.100623)
Supplement: Supplementary file 2 [file mmc2.docx]

| Supplementary Table 2. Lipid-lowering therapy according to history of myocardial infarction | | | | | | | | |  |  |  |
| --- | --- | --- | --- | --- | --- | --- | --- | --- | --- | --- | --- |
|  |  |  |  | |  |  | |  | | |  |
| Variable | | Prior MI (n = 65) | | No prior MI (n = 79) | | | p value | |  |  |  |
| Statin use,n (%) | | 65 (100) | | 77 (97.5) | | | 0.501 | |  |  |  |
|  |  |  |  |  |  |  |  |  |  |  |  |
| **Amond statin-treated patients, n/N (%)** | |  | |  | | |  | |  |  |  |
| Strong statin | | 54/65 (83.1) | | 40/77 (51.9) | | | **< 0.001** | |  |  |  |
| Moderate-intensity statin | | 7/65 (10.8) | | 29/77 (37.7) | | | **< 0.001** | |  |  |  |
|  |  |  |  |  |  |  |  |  |  |  |  |
| Ezetimibe use, n (%) | | 43 (66.2) | | 29 (36.7) | | | **< 0.001** | |  |  |  |
|  |  |  |  |  |  |  |  |  |  |  |  |
| PCSK9 inhibitor use, n (%) | | 5 (7.7) | | 0 (0) | | | **0.017** | |  |  |  |
|  |  |  |  |  |  |  |  |  |  |  |  |
| Ezetimibe and/or PCSK9 inhibitor use, n (%) | | 45 (69.2) | | 29 (36.7) | | | **< 0.001** | |  |  |  |
|  |  |  |  |  |  |  |  |  |  |  |  |
| LDL-C, mg/dL | | 62.0 (47.3-76.5) | | 69.5 (56.0-91.5) | | | **0.017** | |  |  |  |
|  |  |  |  |  |  |  |  |  |  |  |  |
| Total cholesterol, mg/dL | | 139 (113-153) | | 152 (129.5-187) | | | **0.003** | |  |  |  |
|  |  |  |  |  |  |  |  |  |  |  |  |
| Significant p values are written in bold. | | | | | | | |  | | |  |
| Data are expressed as median (interquartile range), or n (%). | | | | | | | | |  |  |  |
|  |  |  |  | |  |  | |  | | |  |
| LDL-C, low-density lipoprotein cholesterol; MI, myocardial infarction; PCSK9, proprotein convertase subtilisin/kexin type 9. | | | | | | | | |  |  |  |
|  |  |  |  |  |  |  |  |  |  |  |  |
