# Supplementary material for: Aortic plaque morphology and cholesterol crystal release assessed using non-obstructive general angioscopy
Source: Atheroscler Plus. 2026 Nov 7;66:100623. doi: 10.1016/j.athplu.2026.100623 (PMC13770710; doi:10.1016/j.athplu.2026.100623)
Supplement: Supplementary file 3 [file mmc3.docx]

| Supplementary Table 3. Mixed-effects logistic regression analysis of site-level cholesterol crystal detection according to plaque morphology | | | | |
| --- | --- | --- | --- | --- |
|  |  |  |  |  |
| Plaque morphology vs PCR | | Odds Ratio | 95% CI | p value |
| PR | | 0.26 | 0.11–0.62 | **0.003** |
| Thrombus | | 0.04 | 0.005–0.31 | **0.002** |
| Ulcer/fissure | | 0.03 | 0.004–0.22 | **< 0.001** |
| Yellow plaque | | 0.02 | 0.003–0.16 | **< 0.001** |

PCR, puff chandelier rupture; PR, puff rupture; CI, confidence interval.

Odds ratios were estimated using mixed-effects logistic regression with a patient-specific random intercept to account for within-patient clustering. Puff chandelier rupture was used as the reference category.
